# Using experience-based co-design to develop mobile/tablet applications to support a person-centred and empowering stroke rehabilitation

**DOI:** 10.1186/s40900-023-00472-z

**Published:** 2023-08-24

**Authors:** Mille Nabsen Marwaa, Susanne Guidetti, Charlotte Ytterberg, Hanne Kaae Kristensen

**Affiliations:** 1 Department of Physiotherapy Education, University College Southern Denmark, Esbjerg, Denmark; 2 Department of Neurobiology Care Sciences and Society, Karolinska Institutet, Huddinge, Sweden; 3 Center for Innovative Medical Technology, Department of Clinical Research, University of Southern Denmark, Odense, Denmark; 4 Women’s Health and Allied Health Professionals Theme, Karolinska University Hospital, Stockholm, Sweden; 5 Geriatric Research Unit, Department of Clinical Research University Hospital Odense, University of Southern Denmark, Odense, Denmark

## Abstract

**Aim:**

To investigate and describe the process of using experience-based co-design (EBCD) to develop mobile/tablet applications to support a person-centred and empowering stroke rehabilitation.

**Setting:**

Two cross-sectoral stroke rehabilitation settings in Denmark comprising six rehabilitation units.

**Participants:**

Stroke survivors (n = 23), significant others (n = 18), occupational therapists (n = 12), physiotherapists (n = 9), representative of a patient organization (n = 1), application developers (n = 3) and researchers (n = 2).

**Method:**

A structured, facilitated EBCD approach comprising six stages was used to co-design a service that aimed to address the priorities and needs of all relevant end-users. Data were collected by interviews, participant observations, notes on “flip sheets” and written feedback on the content in the apps and on the instruction pages. Data were analyzed descriptively and with a constructivist grounded theory analysis.

**Results:**

The content in the application solutions “Mit Sygehus” and “Genoptræn.dk” were co-designed to support the needs identified by all end-users. Relevant evidence-based knowledge, person-centred exercises and guidelines using video recordings were the most important among the developed content in the applications to support person-centred and empowering stroke rehabilitation. Furthermore, easy, and seamless communication were considered important.

**Conclusions:**

EBCD facilitated the development of content in the applications to support a person-centred and empowering stroke rehabilitation. Participants experienced that their contribution was considered important and valued.

**Supplementary Information:**

The online version contains supplementary material available at 10.1186/s40900-023-00472-z.

## Abstract (plain language summary)

Many app-based solutions exist to support stroke rehabilitation; however, stroke survivors and significant others are seldom involved in the design of these solutions, which often reduces the uptake in clinical setting.

By involving stroke survivors, significant others, therapists from diverse rehabilitation settings, patient organizations, app-designers, and researchers, we co-designed the content in two app-solutions to support a person-centred and empowering stroke rehabilitation, based on the needs and wishes of all stakeholders. Stroke survivors and significant others experienced that their voices were heard throughout the co-design process, and that their contribution was considered important and valued.

**Supplementary Information:**

The online version contains supplementary material available at 10.1186/s40900-023-00472-z.

## Implications

- Relevant content of applications to support a person-centred and empowering stroke rehabilitation can be identified and developed using EBCD.
- In depth understanding of stroke survivors, significant others and therapists needs and perspectives are valuable and important in an EBCD process to promote development of new useable solutions.
- Stroke survivors, significant others and therapists perceived that participation in an EBCD process and sharing experiences were valuable.

## Introduction

Stroke is recognized as a major cause of disability worldwide [1] and is often associated with long-term physical, cognitive, social and emotional disabilities [2–4], that can result in dependence and reduced participation in everyday life [3, 5, 6]. To respond to these challenges, priority must be given to person-centred and empowering rehabilitation strategies [7, 8]. It is suggested that empowerment can be achieved through person-centred rehabilitation [9, 10], provided in collaboration between stroke survivors and health professionals, based on the needs, preferences and goals identified by the stroke survivors and their significant others [7, 8]. There are several synonyms for person-centred initiatives, such as client-centred, user-centred and individual focus [11]. In this study we describe person-centred rehabilitation as initiatives/interventions that supports the individual stroke survivors’ and significant others’ needs, preferences and goals for the rehabilitation e.g., therapy, interventions, exercises, and knowledge [7, 8]. Furthermore, a person-centred rehabilitation should start as early as possible, be coherent (i.e., coordinated with smooth transitions between different rehabilitation settings) and should contain goal setting, intervention/training, support for social participation, discharge support and continuous evaluation [7, 12].

Nonetheless, studies show that stroke survivors, significant others, occupational therapists (OTs) and physiotherapists (PTs) working in stroke rehabilitation often experience challenges related to cross-sectoral information- and communication, and gaining an overview of the stroke rehabilitation process and call for a more person-centred and tailored stroke rehabilitation [5, 6, 13, 14]. Several studies suggest that information and communication technologies (ICT) such as mobile phone and tablet applications (apps) have the potential to support person-centred stroke rehabilitation and empower stroke survivors and significant others throughout the rehabilitation process. ICT and apps (e.g. phone calls, messages, reminders, remote monitoring or intervention, videos and images) may support the delivery of relevant, evidence-based health information [15–17], support continuity and reduce gaps in the rehabilitation process [18], and support the feeling of being connected with the therapists [17]. Furthermore, stroke survivors’ motivation, engagement and empowerment to independently perform person-centred exercises post-discharge [17, 19], and to become more active in managing their own health may be supported by ICT and apps [15–17, 20–24]. However, because of the lack of tailoring to individual needs, many app solutions do not support person-centred rehabilitation [13]. Furthermore, lack of end-user involvement in developing the solutions reduces the uptake [25, 26]. A recent scoping review also shows that many app solutions developed to support stroke rehabilitation are narrowly focused on only one or few parts of the entire rehabilitation process e.g., information, exercises, or single health related measurements (weight loss, or blood glucose management), and do not support a person-centred and empowering stroke rehabilitation for stroke survivors and significant others [27].

There is a need to involve end-users in the development of content in the app solutions. “Mit Sygehus” [in English, My hospital] is a knowledge-based and safe app solution implemented in all hospitals in the southern region of Denmark. “Mit Sygehus” is currently implemented in over 300 diagnoses in several departments (e.g. gynecology, oncology, orthopedics, surgery, endocrinology, rheumatology, etc.) [28]. The existing modules in the app have shown potential to accommodate many of the above-mentioned unmet needs expressed by stroke survivors, significant others, and therapists in relation to the stroke rehabilitation process, however, “Mit Sygehus” has not yet been designed, and thus has no content, to support stroke rehabilitation. “Genoptræn.dk” [in English, Rehab.dk], is a training-based app-solution already integrated with “Mit Sygehus” as a “training” module. “Genoptræn.dk” already contains more than 700 generic video-recorded exercises, that can be assigned by the therapists to the patients’ personal app [29]. “Genoptræn.dk” is currently being used in both hospital and municipal rehabilitation settings; however, the content in the app has not yet been developed to support person-centred-stroke rehabilitation.

Stroke survivors represent a heterogeneous group and often have complex needs for person-centred rehabilitation processes. Therefore, stroke survivors, significant others, therapists, app developers and researchers all need to be involved in a co-design process using some of the modules in “Mit Sygehus” and “Genoptræn.dk” and in developing new functions to match the needs of the end-users.

### Experience-based co-design

Patient-involvement to improve health care has been applied since 1993 [30], and experience-based co-design (EBCD) since 2006 [31]. However, EBCD literature within stroke rehabilitation is lacking, and have mainly been focused on improving the activity level of stroke survivors in acute care settings [32–34]. EBCD has often been used as a quality improvement methodology [35], to address the priorities, needs, and wishes of all relevant end-users in a collaborative process [31, 36]. EBCD approaches draw upon participatory action research (AR) design and user experience design [25, 31]. AR principles support the co-design actions in EBCD and recognize that especially qualitative data may more often lead to actions taken to improve patient experiences and healthcare services [31], since quantitative methods do not indicate what needs to be done to improve any situation [25]. EBCD represents a shift in the perception of patients and significant others—from a role characterized by passivity and dependency to participation as more active, empowered and autonomous individuals [31, 35, 37]. The novelty of EBCD is thus involving end-users throughout all research phases and reflecting on the actions and activities from each phase to support the next [25, 35]. Ideally, this results in greater uptake of the service and increased engagement throughout the process [25, 35], provided that equal power dynamics are achieved between stakeholders [30, 32, 38]. There has been limited scientific evaluation of the process and outcomes of the EBCD approach [39], especially within stroke rehabilitation [32]. Therefore, the overall aim of this study was to investigate and describe the process of using EBCD to develop the content in two mobile and tablet apps to support a person-centred and empowering stroke rehabilitation. This included describing how user involvement was achieved and perceived by participants throughout the EBCD process and how the reflections and learnings from each stage of the EBCD cycle supported the actions in the next stage.

## Methods and results

### Experience-based co-design

EBCD involves a structured, facilitated two-phased process that comprises six stages (see Table 1). The discovery phase of EBCD involves the first three stages: setting up the project (stage 1), observations of a particular rehabilitation pathway and engaging patients, significant others, and staff concerning their experiences of rehabilitation (stage 2 and 3). The co-design phase also comprises three stages. In this phase, end-user experiences are analyzed to identify “emotional touchpoints” in healthcare services, where something could have been done better, or which exemplify a good experience. Identified “touchpoints”, supported by existing evidence on the topic, are then presented to all end-users to trigger discussion about local quality issues and agree on a set of improvement priorities, through co-design groups (stage 4). “Touchpoints” can be presented by using “trigger films” or “touchpoint lists”, experience maps, list of improvements or interview quotes [38]. Smaller co-design working groups (stage 5) and a celebration event brings the co-design phase to an end (stage 6) [37]. Reflective questions such as “what did we learn from this stage”, “how was the power relation between participants and how can we make it more equal” and “how useful was this stage for the research process” support the next stages and actions in the EBCD process [25, 35]. The GRIPP2 reporting checklist on patient and public involvement in research was reported in Additional file 1 [40].

**Table 1:** Overview of purposes and activities in the EBCD process

	EBCD phases	Activities		Purpose
Discovery phase	*Stage 1*	*Engaging stroke rehabilitation settings (n* = *6)*		Engage different stroke rehabilitation settings
	Setting up the project 2014–2017	Acute stroke units (n = 2)		
		Subacute stroke units (n = 2)		
		Stroke units in the municipalities (n = 2)		
	*Stage 2* + *3*	*Interviews (focus group or individual)*		Explore experiences of stroke rehabilitation across sectors, identification of unmet needs and use of ICT in the rehabilitation process and everyday life
	Engaging stroke survivors, significant others, and therapists 2014–2017	Stroke survivors (n = 18)		
		Significant others (n = 13)		
		PTs (n = 4)		
		OTs (n = 5)		
	2021	*Observations of stroke rehabilitation settings (5 settings)*		
Co-design phase	*Stage 4*	*Workshop 1 (Jutland) (n* = *14)*	*Workshop 1 (Funen) (n* = *11)*	Present “emotional touchpoints” and the modules in “Mit Sygehus”. Additionally, to generate concrete input on the content of each module, to meet stakeholders’ needs and to prioritize the modules
	Co-design meetings 2021	Stroke survivors (n = 2)	Stroke survivors (n = 2)	
		Significant others (n = 2)	Significant others (n = 2)	
		PTs (n = 3)	PTs (n = 2)	
		OTs (n = 3)	OT (n = 1)	
		App developers (n = 2)	App developers (n = 2)	
		Researchers (n = 2)	Researchers (n = 2)	
		*Scoping review on existing app solutions to support stroke rehabilitation*		
	*Stage 5*	*Step 1: Co-design meetings in Jutland working with “knowledge” module (n* = *4)*	*Step 1: Co-design meetings on Funen working with “knowledge” module (n* = *3)*	Co-design content in “Mit Sygehus” to meet stakeholders’ prioritized needs. Furthermore, to assess if the “training module” was suitable to meet stakeholders’ needs and applicable in the different rehabilitation settings
	Small co-design meetings 2021	PT (n = 1)	PT (n = 1)	
		OT (n = 1)	Speech therapist (n = 1)	
		Head therapist (n = 1)	Physician (n = 1)	
		Representative from patient organization (n = 1)		
		*Step 2: Co-design meetings in Jutland working with “training” module (n* = *4)*	*Step 2: Co-design meetings on Funen working with “training” module (n* = *5)*	
		PT (n = 1)	PT (n = 1)	
		OT (n = 1)	OT (n = 1)	
		App developer (n = 1)	App developers (n = 2)	
		Researcher (n = 1)	Researcher (n = 1)	
	*Stage 6*	*Workshop 2 (Jutland) (n* = *10)*	*Workshop 2 (Funen) (n* = *9)*	Presentation of app solution and “hands-on” test of all the content in the app, as well as feedback on written instructions to be used in later a testing period
	Celebration event 2021	PTs (n = 2)	Stroke survivor (n = 1)	
		OTs (n = 3)	Significant other (n = 1)	
		Representative from a patient organization (n = 1)	PT (n = 1)	
		App developers (n = 2) -one attended online	OTs (n = 2)	
		Researchers (n = 2)	App developers (n = 2)	
			Researchers (n = 2)	
			*Workshop 2 (Funen) (n* = *4) Online*	
			PT (n = 1)	
			OT (n = 1)	
			App developer (n = 1)	
			Researcher (n = 1)	

### Discovery phase

#### Stage 1: setting up the study

In Denmark stroke rehabilitation may take place in different stroke rehabilitations settings. Initially, the stroke survivor is admitted to an acute hospital stroke unit. When required, rehabilitation may continue in a sub-acute hospital stroke unit and/or rehabilitation continues in the municipalities (i.e., home-rehabilitation or in rehabilitation centres). Participants in need of rehabilitation in all rehabilitation settings most often have more complex difficulties (i.e. cognitive, emotional, mental and/or physical) and need individualized and intensive interdisciplinary rehabilitation delivered in acute and subacute stroke units, before they can continue their rehabilitation in the municipalities [41]. The rehabilitation process when in need of all three rehabilitation settings may range from 1 to 4 months. To have representation from all stroke rehabilitation settings, two acute stroke units, two subacute stroke units (i.e., the hospitals responsible for in-ward stroke rehabilitation in the region of Southern Denmark) and two larger stroke rehabilitation units in the municipalities were engaged in stage 1.

#### Stage 2 and 3: engaging patients, significant others, and staff and performing observations

##### Participants

The head therapist of each rehabilitation setting identified two therapists (one OT and one PT) who voluntarily agreed to participate in focus group interviews in this stage. Stroke survivors living at home who had just ended their rehabilitation process throughout all rehabilitation settings (i.e., participants with more complex difficulties) and significant others (i.e., someone who was close to the stroke survivor and offered support) were identified by the municipal therapists to participate in interviews in this stage. The inclusion criteria for stroke survivors were (a) ability to understand and answer interview questions and (b) experience of ICT before and after stroke. For therapists, criteria were that they should (a) work in one of the stroke rehabilitation settings, (b) as either a PT or OT.

##### Method

To capture experiences in relation to the stroke rehabilitation process, unmet needs, and the integration of ICT as part of the rehabilitation process and in everyday life, individual or focus group interviews were held with stroke survivors (n = 18), significant others (n = 13), and therapists (n = 9) from different settings of the Danish stroke rehabilitation process. Stroke survivors and significant others were interviewed 6–12 months post-stroke. Analysis of the transcribed interviews was performed using a constructivist grounded theory (GT) approach comprising open and focused coding, constant comparative method and theoretical sensitivity [42].

Additionally, prior to stage 4, non-participant and participant observations were performed by the first author in five of the six stroke rehabilitation settings included in this study for a total of five full days. The observations had several purposes and benefits: (1) as an “outsider” in all rehabilitation settings, the obvious benefit was that relations and connections were made with all participating therapists, (2) therapists used the opportunity to ask more about the project and get a greater insight to the purpose of the next stage of the research process, and (3) observations supported and contributed to the identified “touchpoints”. The municipality in Jutland agreed to participate in this study just before stage 4 of the EBCD process, and therefore there was not time enough to perform observations in this municipality.

Insight to similarities and differences in local organizational processes, context, interventions, and interactions among therapists, stroke survivors and significant others was valuable knowledge.

For example, knowledge regarding the short hospitalization and the number of therapists involved in the stroke survivors’ rehabilitation process made it clear that especially in acute rehabilitation settings continuity was difficult to achieve, which may challenge the implementation of the apps. The insights from the observations were also used to facilitate discussions in the workshops, regarding which apps and which content that would fit the different settings.

##### Findings

The results from the interviews have been reported elsewhere [5, 6, 13]. In summary, findings indicated that stroke survivors and significant others welcomed the use of ICT and apps to support stroke rehabilitation, since they may promote activity, participation in everyday life, independency, and adherence to perform person-centred exercises [5, 6]. Therapists highlighted that ability to use ICT and apps are crucial for stroke survivors in today’s society and that ICT and apps may support continuity and coherence of the rehabilitation process as well as support a person-centred rehabilitation e.g., through videorecorded exercise therapy and guidelines [13].

A cross-analysis was furthermore performed by the first author (and discussed with the last author), using a constructivist GT analysis to find “emotional touchpoints” across interviews to inform stage 4. The purpose of a cross-analysis was to make sure to bring participants’ experiences and voices to the next research stage and to transform these into actions.

### Co-design phase

#### Stage 4: co-design meetings

##### Workshop 1

*Purpose* The purpose of the first workshops was to generate concrete input on the content of each module of the apps, to meet the needs of all participants and to prioritize the modules. Additionally, to understand which mechanisms in stroke rehabilitation that were perceived important by the stakeholders to support empowerment and how participating in the EBCD process was perceived.

The workshops were planned and led by the first and last author in the spring of 2021 and took place in the Region of Southern Denmark (one in Jutland, the western part of Denmark, and one on Funen, the central part of Denmark).

*Participants* The head therapists (mentioned in stage 1) identified one OT and one PT from each rehabilitation setting to participate in the workshops. Therapists from the municipalities identified stroke survivors living at home that had either recently terminated or were about to terminate their rehabilitation process and had received rehabilitation in all rehabilitation settings (acute, subacute and rehabilitation in the municipalities, i.e., having complex difficulties, however being able to participate for 2 h and without severe communication deficits) and a significant other to participate in the workshops. In the Jutland workshop, 14 participated and 11 on Funen (see Table 2 for participant characteristics). Of the 12 therapists participating in stage 4, three had also participated in the interviews in stage 2 and 3. Each workshop lasted for 2 h.

**Table 2:** Characteristics of participants in the two workshops

Participants	Age, years	Gender	Origin	Workshop 1	Workshop 2	Rehabilitation setting	Work experience in neurological rehabilitation, years
Stroke survivor and husband	63/66	Female/male	Funen	X			
Stroke survivor and wife	85/79	Male/female	Funen	X			
Stroke survivor and wife	57/55	Male/female	Funen		X		
Stroke survivor and cousin	37/35	Female/female	Jutland	X			
Stroke survivor and husband	69/74	Female/male	Jutland	X			
OT	44	Female	Funen		X	Acute	18
OT	47	Female	Funen		X (online)	Subacute	19
OT	38	Female	Funen		X	Municipality	10
OT	46	Female	Funen	X		Municipality	13
OT	46	Female	Jutland	X	X	Acute	4
OT	29	Male	Jutland	X	X	Subacute	6
OT	36	Female	Jutland	X	X	Municipality	3
PT	35	Female	Funen	X	X (online)	Subacute	11
PT	37	Female	Funen	X	X	Municipality	6
PT	43	Female	Jutland	X		Acute	14
PT	25	Female	Jutland	X	X	Subacute	2
PT	36	Female	Jutland	X	X	Municipality	8
Representative from patient organization	48	Female	Jutland		X		

*Method* The workshop was started by participants giving written informed consent to participate, and consent to allow photographs to be taken during the workshops that could be used for presenting the results. Prior to attending the workshop, the participants had received written information about the project and the purpose of the workshops. The first author introduced the background for conducting the workshops. “Emotional touchpoints” identified from stage 2 and 3 (Table 3) were also presented i.e., the need for easier access to knowledge about stroke and the option to have the text read out loud. Furthermore, the need for more person-centred rehabilitation was presented, i.e., exercises that would be tailored to a person’s specific motor and/or cognitive difficulties, video-recorded guidelines for transfers, etc. Also presented were the need to minimize the gaps when a patient transfer between rehabilitation settings, through easier documentation and communication with significant others and colleagues across rehabilitation settings. Other important “touchpoints” presented were a need for follow-up when rehabilitation terminates, continuous support from therapists throughout rehabilitation and support to learn to use ICT and apps that may support rehabilitation and everyday life, thereby relieving strains on significant others. Also, a need for support to establish contact with peers/support groups was presented. Accommodating the expressed needs identified in Table 3 was intended to lead to a more person-centred stroke rehabilitation and increased empowerment in stroke survivors and significant others [7, 9, 10, 12].

**Table 3:** Emotional touchpoints

	Emotional touchpoints—Needs and areas for improvement identified			
	Acute and subacute care in specialized stroke units (inpatient) Typically, 0–4 weeks	Transition to home-rehabilitation	Rehabilitation in the municipalities Typically, 1–3 months	Maintenance phase (rehabilitation terminated)
Stroke survivors	Easy access to relevant information (diagnosis, prognosis, and consequences (brochures, app solution) and “read out loud” function An overview of the goals and planned activities (rehabilitation plan) Assessments of the ability to use ICT and need to learn to use ICT (mobile phone, tablet, computer, apps) Need for person-centred guidelines and exercises	Need for overview and transparency	Managing everyday life (learning what deficits influence daily living and participation) Tracking (phone calls to feel safe/physical activity tracking) Need for support to learn to manage ICT Staying connected through ICT	Access to health professionals continuously (new questions arise, a need for answers) Support groups (peers)
Significant others	Easy access to relevant information (diagnosis, prognosis, and consequences (brochures, app solution) and “read out loud” function Need for insight into rehabilitation plan Need for therapists to support person-centred daily training (relieving the significant others)	Involvement No gaps Skills to manage new role	Insight into rehabilitation plan Therapists to support daily training (relieving the significant others) Stroke survivor being able to manage ICT—training/skills (to feel safe/being able to participate in daily activities, gaining overview (work, hobbies, etc.)	Lack of follow-up A “life-line” to reduce strain (responsibility shared with health professionals regarding adherence to exercises/activities)—reminders, phone calls, text messages, app solution) Peers to share experiences with
Therapists	Assessment, goal setting and training of cognitive and physical deficits/resources (using ICT when relevant) Teaching skills to manage ICT and how to comprehend/get an overview of a complex rehabilitation process Easy and simple ICT solutions for stroke survivors Person-centred guidelines for transfers, exercises, and daily activities (pictures, documents, videos, “read out loud” function) Easier documentation/communication in and across sectors (also with significant others)	Sharing information across sectors (pictures, videos) Different IT systems in sectors is a barrier Fewer places to document	Training gross motor skills, fine motor skills and cognitive deficits Involving caregivers even more Learning to manage new life conditions—using ICT to compensate (calendar, reminders, dictate messages)	Monthly/3-monthly phone calls from health professionals Access/contact to patient organizations (peers)

Additionally, knowledge from a recent scoping review on existing apps used to support stroke rehabilitation [27] supported the list of “touchpoints” and the content of the workshops. The review showed that app solutions to support stroke rehabilitation can be used in different rehabilitation settings, however most existing app solutions only support a limited aspect of the rehabilitation process, e.g., assessment or training, and do not accommodate end-users’ need for more comprehensive person-centred solutions.

**Fig. 1:**
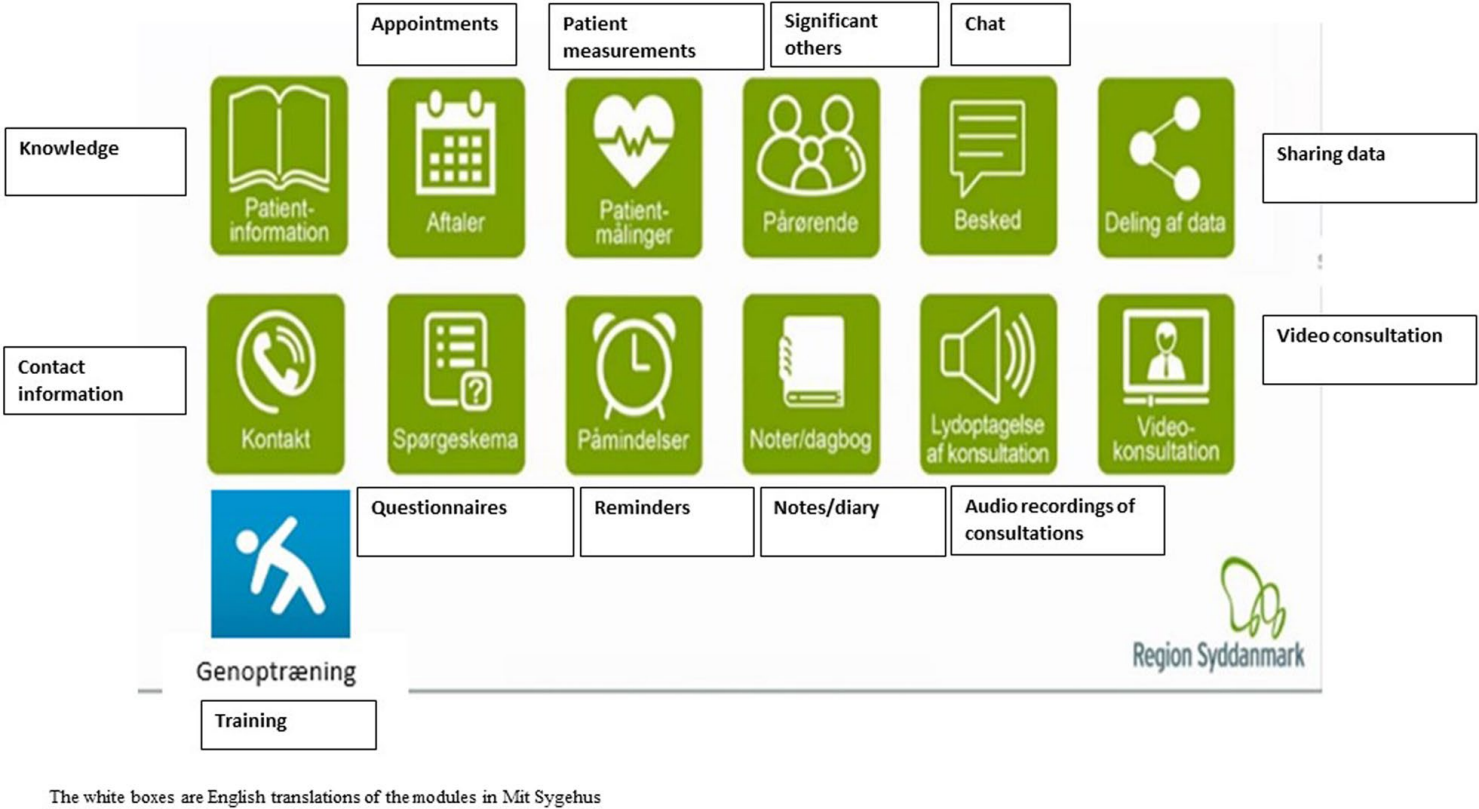
Available modules in Mit Sygehus. The white boxes are English translations of the modules in Mit Sygehus

Next, participants were given an insight into existing modules in “Mit Sygehus” (see Fig. 1). “Mit Sygehus” encompasses 12 different modules: (1) knowledge module (evidence-based information about the diagnosis, treatment, rehabilitation etc.), (2) an overview of all appointments, (3) patient measurements (e.g., weight, blood pressure, etc.), (4) significant others (i.e., information that is specifically important for significant others, such as peer support groups), (5) communication with health professionals through a chat function, (6) sharing patient data with health professionals, (7) contact information for relevant rehabilitation settings, (8) option to fill out relevant data or questionnaires prior to consultations, (9) reminders, (10) personal notes/diary, (11) audio recordings of consultations, and (12) video consultation providing significant others, cross-sectoral colleagues or the patient themselves the opportunity to participate in consultation without being present at the hospital. These modules are to be seen as headlines without any content, yet.

“Genoptræn.dk”, which can work as an integrated “training” module in “Mit Sygehus”, was also included for this study, to meet end-users’ need for easy access to all relevant content related to the stroke rehabilitation in one place [13]. However, “Genoptræn.dk” only contain generic exercises. Therefore, to accommodate earlier identified needs for more person-centred exercises and guidelines [5, 6, 13], prior to the workshop, a “video recording” function was developed. The intention of this “video recording” function was that therapists during interventions, recorded for example specific exercises while verbally supporting and guiding the stroke survivor. The person-centred recordings are then to be stored safely in the stroke survivors’ personalized app solution serving also as documentation and communication within and across rehabilitation settings. If the stroke survivor consents, significant others have the same access to the contents of the app.

**Fig. 2:**
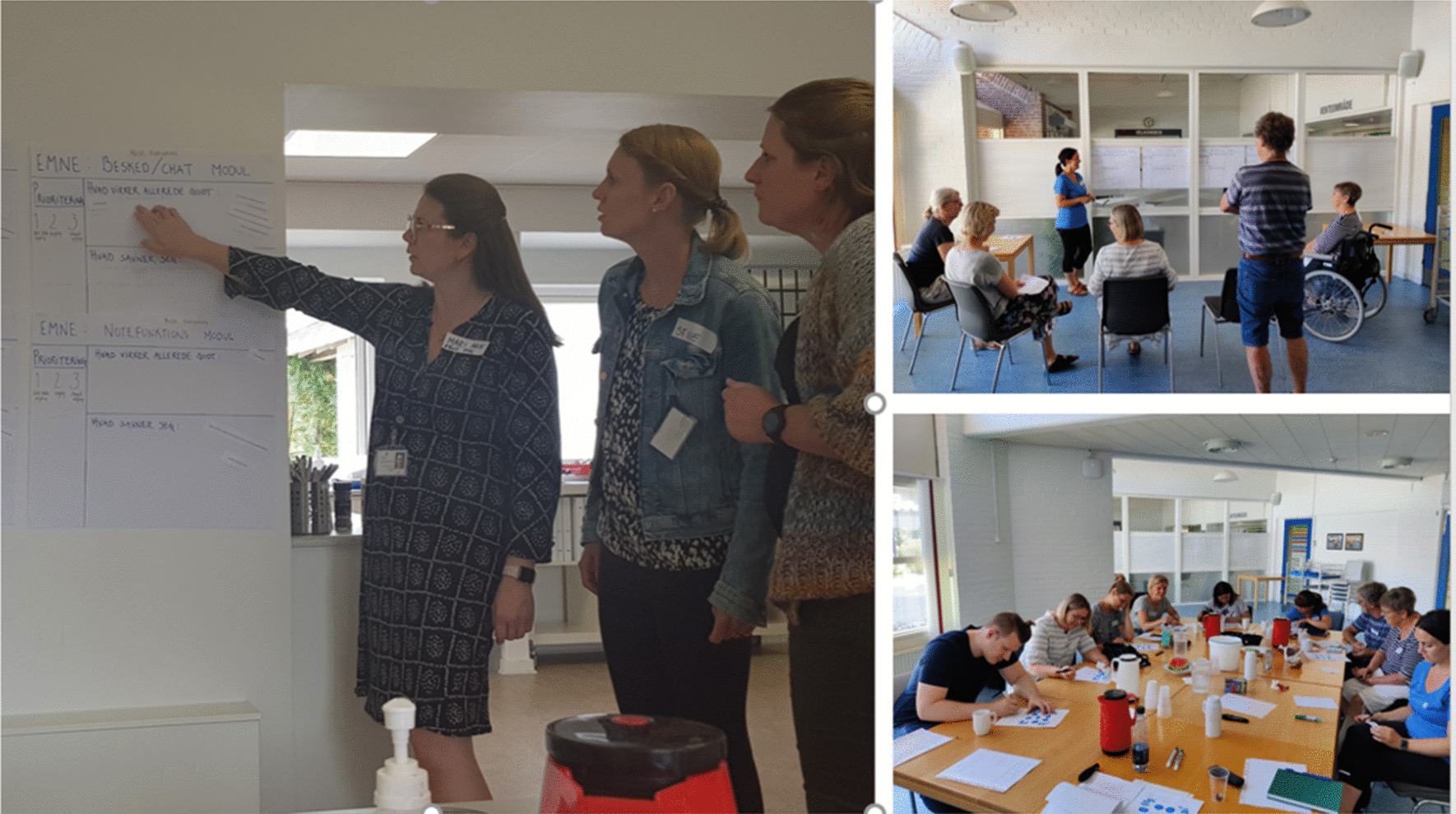
Input on the content in “Mit Sygehus” and prioritizing modules using “flip sheets”

The workshop was then divided in two stations in each end of the room, and participants were split into two groups. After consulting with the therapists, the stroke survivors and significant others were paired with a therapist they knew, to create a safe space where they would feel comfortable and motivated to equally contribute to the workshops [43]. Each workshop comprised three or four big “flip sheets” with seven modules from “Mit Sygehus”: “knowledge”, “training”, “reminders”, “chat”, “contact information”, “notes/diary”, and “video consultation/audio recordings of consultation”. Statements/suggestions from the identified “touchpoint” were added to each headline (as small written sentences), to support the discussions in the workshops (see Fig. 2 and Table 4). The module’s “appointments” and “significant others” were integrated in the “knowledge” “flip sheet”. The activities at each station lasted for 30 min, with a 15-min break in between.

**Table 4:** Input on the content and prioritizing the modules

Modules discussed in workshops	Input on the content in the modules (n = 17)	Grade 1: not important	Grade 2: important	Grade 3: very important	Not reported
Knowledge module	Stroke information and treatment Information about cognitive deficits The rehabilitation process and phases The maintenance phase (phase 4) Support group/peers Daily appointment/programme Video/audio of the information			17	
Training module	Step-by-step guidance about transfers, daily activities—also as communication Personalized video recordings of exercises and progress Easily accessible Available when rehabilitation determines	1	5	10	1
Reminder function	Reminders to exercise and appointments Must be personalized	2	2	5	8
Chat module	Worried that it will escalate Who will answer the questions and when? How long is this option open?	5		1	11
Note/diary module		3			14
Contact module	Pictures of staff Contact information for all relevant departments and treating staff		1	7	9
Video consultation	Participation from other stakeholders (social worker, coordinator, significant others, therapists from other rehabilitation setting, etc.) Video consultation/follow-ups better than phone calls	2	7	7	1

The first and last author who attended the workshop, made individual observational notes.

*Findings* Lessons learned from the first workshops were that presenting the “touchpoint” analysis from earlier studies had a validating effect, since participants agreed on the needs identified. They were also asked to supplement the list of “touchpoints” on the flip sheets. Several inputs to make the “training” module more useful were identified. Also, recordings of the stroke survivors’ current functioning were mentioned, to make it easier to follow progression for all end-users:

I would have liked that access to the exercises (significant other, Funen)

Insight to what exercises and the stroke survivors’ abilities before arriving here (PT, municipality rehabilitation setting, Funen)

One stroke survivor furthermore pointed out that exercise therapy through video recordings should be available also after rehabilitation has ended in the municipality to continuously support adherence. Additionally, a PT suggested that providing relevant person-centred information through an app could support coping with the new situation stroke survivors and significant others find themselves in:

Relevant information may facilitate coping (PT, subacute rehabilitation setting, Funen)

Each end-user also graded each module to be: (1) not important, (2) important, or (3) very important after working on each station (see Table 4). For example, the “diary” module was graded “not important” by three participants while 14 did not grade this module and the “chat” module was only graded important by one therapist. The “training” module was graded “very important” by most participants, especially because it included the chance to make exercises more person-centred than was the case in existing known solutions. Also, the idea that gait function, transfer guidelines or instructions for daily activities could be recorded and stored as documentation and shared for communication was highly welcomed and considered to be necessary for person-centred and empowering stroke rehabilitation. Significant others were also excited about the idea that exercise support was provided through the app, with reminders as additional support (see Table 4).

##### Empowerment exercise

*Method* As a final exercise, based on theory and literature on empowerment [9, 10, 44, 45] participants marked on an empowerment model (constructed by the first author) which key elements they felt were important for their experience of empowerment in stroke rehabilitation. The model of empowerment contained six headings with supporting sentences: (1) communication (to and between health professionals and rehabilitation settings), (2) knowledge (about stroke, prognosis, rehabilitation plan), (3) support from health professionals, (4) involvement (in goal setting and in rehabilitation plan and activities), (5) control and overview, and (6) mastery (to act on their own, to participate in valued activities, to find the knowledge needed).

*Findings* The results from this exercise indicated that mechanisms such as better communication between health professionals (e.g., on the stroke survivors’ goals, progress, and status) when a patient transfer between rehabilitation settings, is important to experience empowerment. Sufficient knowledge about stroke and the rehabilitation plan, active involvement and greater overview of own rehabilitation, and skills to participate in valued activities in everyday life were also mentioned as important mechanisms. Additionally, most therapists wrote elaborating sentences, stating that they considered individualizing the rehabilitation to the needs of stroke survivors and involving them would be empowering. Participants were also asked to give written feedback on participating in the workshop, and all participants valued it as a great experience. Therapists found it rewarding to get an insight to greater part of the rehabilitation process from the perspectives of stroke survivors and significant others, and to discuss the rehabilitation content with colleagues in other rehabilitation settings.

It has been rewarding to get knowledge about the experiences from the patient and significant other related to the entire rehabilitation process and to have the opportunity to discuss this with cross-sectoral colleagues (OT, subacute rehabilitation setting, Funen)

One PT even suggested that more stroke survivors and significant others could have been included to get more perspectives. Stroke survivors and significant others were positively surprised about the opportunities in the apps, and on how their experiences and opinions were valued in the workshops. Also, they appreciated the opportunity to refresh their rehabilitation experiences.

I was surprised to see the many opportunities in the apps, and I felt that the others listened to what I said (stroke survivor, Jutland)

##### Reflections on the first workshops

Data analysis from participants’ inputs on “flip-sheets”, and their prioritization, facilitated the discussion on how to proceed to the next stage of EBCD.

In the workshops all participants’ experiences and views were valued by each other. Bearing in mind that both researchers (respectively trained PT and OT) had not been working in stroke rehabilitation settings for several years, open-ended and elaborating questions during facilitation were used. Questions from the therapists and/or the two facilitators (researchers), such as: “what content would have supported you in this module?”, “would you use this module?” and “if we need to learn from this, what would you suggest be done differently” made it possible to show sincere interest in making stroke rehabilitation better for participants and supported their active engagement. Also, being paired with a therapist they knew had a positive effect on sharing their experiences.

To make sure that participants discussed all the modules in both sessions within the time they were given, more facilitation was provided in the workshop on Jutland. Since stroke survivors expressed that attending the workshop for 2 h were cognitively fatiguing, extending the time of the workshops was not an option. Prioritizing the modules was less strenuous than anticipated. Despite different perspectives and experiences participants co-constructed their prioritization of each module. For example, if a stroke survivor initially scored a module “not important”, and the significant other explained the reason for a higher prioritization, they could agree on either disagreeing or changing one of the scores when receiving insights to new perspectives. These argumentations between all participants, showed that there existed an open, equal, and respectful collaboration between participants.

#### Stage 5: small co-design meetings

Small co-design groups worked on the most relevant modules prior to the second workshop.

##### Step 1

Based on participants’ joint priorities, the first author initially worked with the two modules that were prioritized the highest, i.e., the “knowledge” module and “training” module. Relevant evidence-based information was added into “Mit Sygehus” using a “test patient”. Then, access to the app was shared with health professionals from two different sub-acute stroke units, given that longer duration in this rehabilitation setting may give the stroke survivors the time to learn how to use the app. One OT, two PTs, one head therapist, one physician, one speech therapist and one representative from the patient organization “Hjerneskadeforeningen” [in English, the Brain Injury Association] (see Table 1) gave their feedback to the content in the app. Mostly, the feedback implied having more reader friendly language, being more concise in the language choice, giving more examples and having more information on cognitive deficits. Also, the representative from the patient organization suggested creating links to the municipal “brain coordinators” and the “significant other supervisor” (i.e., a health professional that may support significant others) in the app.

##### Step 2

The newly developed recording function in the “training” module was tested in two different stroke rehabilitation settings (subacute stroke unit and municipality rehabilitation) together with two OTs and two PTs. The purpose was to test if the recording function was easy to use for the therapists, to assess the quality of the recordings made and to assess if the time needed to upload the recordings to the patient’s app was acceptable. Both tests revealed the need for smaller adjustments; for example, the duration of the recorded videos was made visible, and all engaged therapists had access to the stroke survivors’ “training” module, to be able to access assigned exercises and adjust content according to current needs of the patient (i.e., person-centred rehabilitation). The app developers and the first author worked with the feedback prior to the second workshop in stage 6. Furthermore, written instructions on how to download the apps “Mit Sygehus” and “Genoptræn.dk”, as well as how to turn on a “read-aloud” function on the mobile phone or tablet were developed, prior to the second workshop.

Step 1 and 2 in stage 5 of the EBCD showed to be valuable for bringing more useable app solutions to the next stage. Especially, the need for written instruction on how to download and use the apps were valuable input to make implementation more successful.

#### Stage 6: celebration event

The last stage of the EBCD process was held as part of the second workshop to recognize the achievements of the co-design process and the participants taking part [33, 37] by introducing the pilot version of the co-designed app solutions. Experiences and feedback were sought from participants at this stage.

##### Workshop 2

*Purpose* The program and purpose for the workshop were presented, which was to test all modules in “Mit Sygehus” using participants’ own mobile phones/tablets and to give feedback on the written instructions to use the app-solutions. For that reason, this workshop had a more practical hands-on focus than the first workshop.

*Method* The second 2-h workshop was planned and led by the first and last author and carried out with participants in Jutland (n = 10) and Funen (n = 9). Additionally, an online workshop was held with two therapists due to high workload and COVID-19-related isolation (see Table 1). None of the stroke survivors and significant others participating in the first workshop (stage 4) were able to attend the second workshop due to other engagements or COVID-19-related isolation. Therefore, the second workshop in Jutland was performed without stroke survivors and significant others; however, a representative of the patient organization participated and spoke on their behalf.

**Fig. 3:**
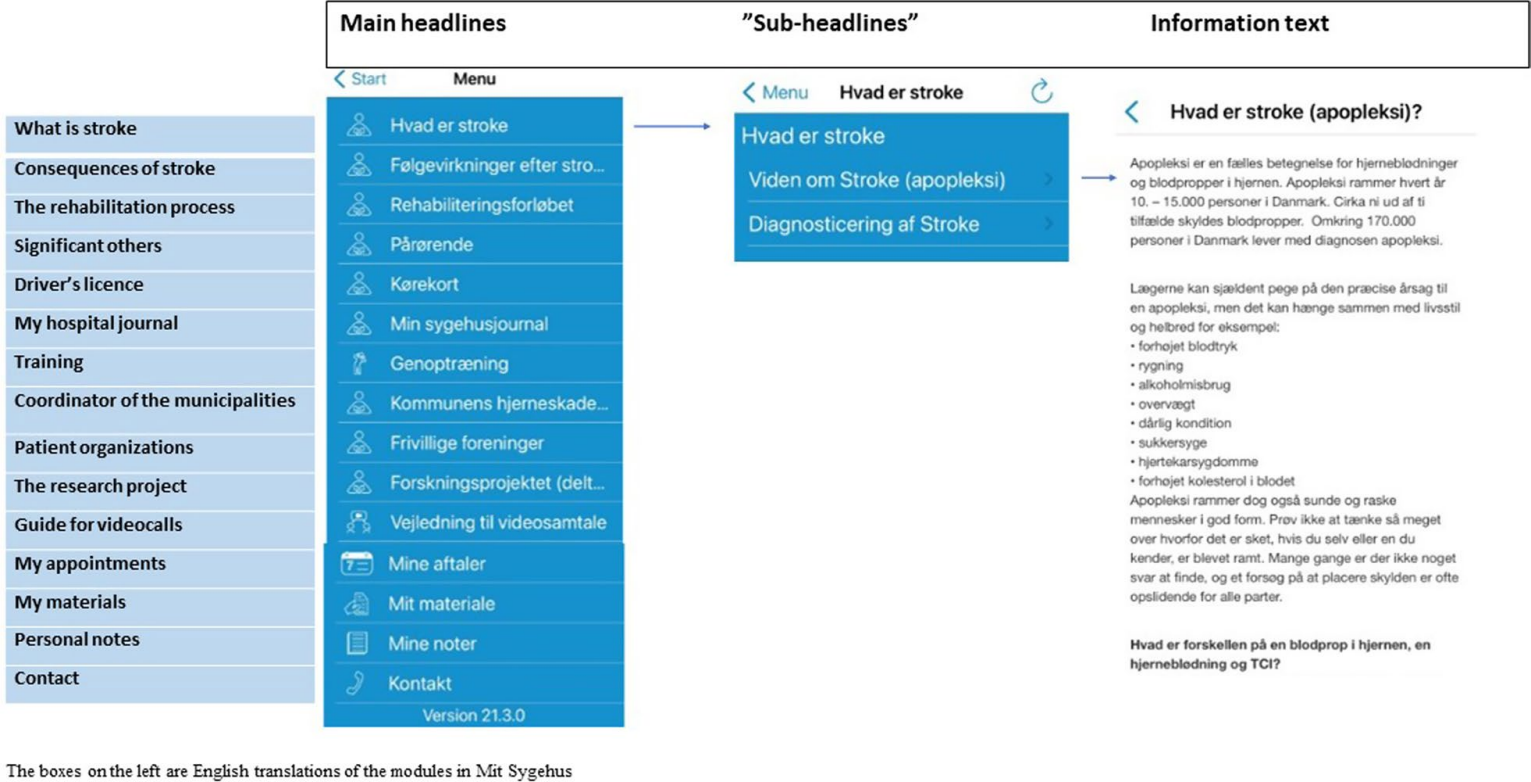
Content of “Mit Sygehus” to support person-centred and empowering stroke rehabilitation

The group was divided into two sub-groups. After consulting with the therapists, the stroke survivors and significant others were paired with a therapist they knew, in the workshop on Funen, to create a safe space where they would feel comfortable and motivated to equally contribute to the workshops. Both groups received written instructions on how to download and use “Mit Sygehus” and on how to install a “read-aloud”-function on their own mobile phone/tablet. They were instructed to visit all modules in the pilot version of the app (see Fig. 3), except the “training” module, which was to be the next task in the workshop. They were also asked to give feedback on the headings and “sub-headings” chosen for each module. They worked with the task for 30 min and, after a 15-min break, they tested the “training” module. Participants were asked to download “Genoptræn.dk” and make a recording of an exercise using an iPad, upload it to the test-patient’s app and watch the exercises on the app afterwards using their own mobile phones/tablets. The participants were asked to give feedback on the written instruction on how to download “Genoptræn.dk” and use of the “training” module.

*Findings* Participants in stage 6 unanimously found the app solutions relevant to support stroke rehabilitation and empowerment. In particular, the option to read/listen to relevant content in the app when needed by the stroke survivor and/or significant other, and the option for person-centred rehabilitation using the “training” module were mentioned as potentially benefitting stroke rehabilitation.

The second workshop only gave minor adjustment to the content of the app solutions and the written instructions. It was suggested to change the heading “consequences of stroke” to “effects of stroke” since this was perceived by the therapists as less confronting and persistent. Changes to the written instruction were mostly about clarifying the different steps in the instruction by using numbers and circles to highlight.

##### Reflections on the second workshop

In the second workshops all participants’ experiences and views were valued by each other. Questions from the therapists, the app developers or the two facilitators (researchers), such as: “What do you think about this module?”, “Could this module have been of importance in your rehabilitation process?” and “Would you use this function?” supported participants’ active engagement. Statements such as: *“This function could have benefitted our rehabilitation”* (significant other, workshop 2, Funen) and *“This function is really great and smart, I would have liked this in my rehabilitation”* (stroke survivor, workshop 2, Funen), also showed how the participants were actively engaged throughout the workshops.

## Discussion

In this study end-users were involved throughout the process of developing the content in two app solutions to enable a person-centred and empowering stroke rehabilitation. In the development process, the participants prioritized the “knowledge” module and “training” module as of greatest importance. Tailored app solutions to meet end users’ needs and support stroke survivors’ deficits were also found important elsewhere [46]. The need for general information and education about stroke, patient organizations, where to find peers, links to relevant webpages, and the option to have text read aloud were also found important in another study [47]. Patient education is a known predictor for increased engagement, satisfaction, and treatment adherence. Given that patients may only remember a limited amount of information delivered in a short time, apps are suggested to be useful in providing education and information that can be accessed as often as liked, at any place and time [48].

Stroke-related deficits are both complex and heterogeneous [41]. The “training” module with the newly developed recording function with audio support allows for exercises and guidelines to be personalized to end-users’ specific needs (e.g., cognitive, speech, facial and motor exercises and/or guidelines for supporting transfers or other daily activities), thus promoting adherence and empowering behaviors. Person-centred exercises/guidelines with audio instructions may be important support tools, especially for stroke survivors who suffer from cognitive or speech-related deficits [47]. Furthermore, the “training” module may support, guide, and inform significant others and colleagues within and across rehabilitation settings. Finally, therapists across rehabilitation settings have the same access to content in the app, allowing them to see, follow-up and adjust the stroke survivors’ exercises, thereby promoting coherent person-centred and empowering stroke rehabilitation. Feedback on exercises to increase motivation and adherence is suggested to support the stroke survivor (e.g., symbols, sounds, etc.) [47]. In the “training module” stroke survivors click “done” when each exercise has been performed; however personalized feedback on performance is not an option. The therapists, however, can access statistics on the exercises that the stroke survivor has/has not performed, and follow-up in the next session, if needed.

The reminder function in “Mit Sygehus” was valued important/very important by seven participants in our study. This potentially empowers stroke survivors and relieve strain on significant others. The reminder function to complete exercises or attend appointments was also valued important in another study [46]. Although not found important in this study, chat functions have been found important to promote communication and a feeling of connectedness to the health professionals from a distance [47], thereby promoting stroke survivors’ confidence, empowerment, motivation and satisfaction with the rehabilitation process [46]. Furthermore, easy access to support from health professionals prevents the experience of being left on their own after discharge from hospital [47]. Digital solutions have been suggested to support stroke rehabilitation through easier access to the rehabilitation plan, goals, and agreements made during consultations, because of the fact that information may be forgotten or misunderstood [47]. This was also mentioned by end-users of the current study, and since much information in the “knowledge” module is of a generic character, it was suggested in the second workshop that individualized plans and agreements could be uploaded in the “training” module, with a heading indicating the content (e.g., goals, status, rehabilitation plan, etc.). This would make it easy for stroke survivors, significant others, and therapists within and across rehabilitation settings to access this information, thus facilitating a person-centred and empowering stroke rehabilitation. Literature about empowerment [9, 10, 44, 45], states that sufficient knowledge, easy and seamless communication within and between rehabilitation settings, overview of rehabilitation plan and person-centred approaches (e.g., involvement in plan and goals, exercises, guidelines etc.) are important to the experience of empowerment, which aligns well with the content developed for the app solutions in our current study.

Several studies have shown that a majority of stroke survivors have positive attitudes towards including ICT and app solutions in the rehabilitation process and everyday life (e.g., exercises, social participation and/or communication) [5, 49–51]. However, to increase acceptability and usability, it is recommended that app solutions to support stroke rehabilitation are easy and simple. This involves limited clicks and limited amount of information. Furthermore, support on how to use ICT and apps has been mentioned as crucial to successful uptake [6, 13, 47, 51]. However, this may be no different from healthy older adults who needs to learn to use ICT or apps [50]. Thus, “Mit Sygehus” and “Genoptræn.dk” are integrated app solutions that provide everything “in one place” for the stroke survivor and the significant other. Also, written instructions were developed, and all participants had “hands-on” in the second workshop, to test the app solutions and to identify the elements that require more support from therapists, when these apps are to be implemented in stroke rehabilitation in a later stage. It is furthermore recommended that new interventions developed through co-design are evaluated upon implementation, and that participants are involved in identifying relevant outcomes [39]. In the final exercise of the second workshop, participants gave their insights on components that are of importance to experience empowerment, which will be valuable when performing an evaluation on the use of the app in the next stage of the research project [39]. The app solutions will be implemented in 8–10 cases (one case being a stroke survivor, a significant other and an OT and PT). As recommended by the Good Practice Guidelines on Health Apps and Smart Devices evaluation on user acceptability and usability [52] and experienced empowerment will be performed in the next phase of this research project. Qualitative interviews (dyadic interviews with stroke survivors and significant others and focus group interviews with therapists) as well descriptive statistics on the use of the app solutions are planned.

### Reflections on EBCD

This study showed that it was possible to use a structured EBCD approach to develop the content in two app solutions to support person-centred stroke rehabilitation (i.e., develop solutions that support stroke survivors’ and significant others’ various needs, preferences, and goals). Despite knowledge on unequal power dynamics in co-production designs [35, 53], statements from the first workshop such as “*it was valuable to receive different perspectives*” (OT, first workshop), “*it has been rewarding to refresh the rehabilitation process*” (significant other) and “*I felt heard*” (stroke survivor) supported that this development process was associated with positive experiences, including learning together and a feeling of being listened to, similar to findings in another study [39]. Sincere interest in lived experiences, creating a safe space to share these experiences and participation of several stroke survivors and significant others may facilitate and motivate stroke survivors and significant others to share both positive and negative experiences in hopes of that other can learn from them [43]. Accomplishing a more equal power relation through the use of EBCD was also seen in a recent review [38]. The results indicate that being engaged in co-design research may have facilitated a sense of accomplishment and empowerment [54]. Another review on experiences of co-design research also found an increase in participants’ self-esteem, and experience of being educated in relation to user participation [30].

We learned that facilitation is important to maintain momentum during the workshops’ different stages, actions and activities, which is supported in previous studies [32, 38]. The implementation and uptake of the solution may have been facilitated by structured user-involvement in all stages and having a “hands-on-focus” in the second workshop, which gave all participants the opportunity to test the written instructions and the pilot version of the apps [25, 55]. Furthermore, the following drivers of importance for developed co-designed solutions to be sustainable have been identified: continuing the work of improving the solutions in each context (i.e., local implementation/quality improvement teams that prompts follow-up actions), mobilization of narratives of being heard and seen, and management systems (i.e., involvement of leaders and decision makers) [25], which is essential knowledge when implementing the app solutions in the next phase of the research project.

### Strengths and limitations

The engagement of end-users throughout the process of developing the content in the two app-solutions to support stroke rehabilitation and empowerment is a strength of this study. One known limitation of EBCD is that it is time-consuming for both end-users and researchers. Furthermore, end-users may be heterogeneous and experience a lack of consensus [25, 55]. However, despite the heterogeneity of end-users involved in this study, all participants agreed that the “knowledge” and “training” module were of greatest importance in the app solutions. The lack of stroke survivors and significant others in the second workshop is a limitation. Finally, even though end-users participated throughout the EBCD process, it is unlikely that all stroke survivors and significant others may benefit from the app solutions, due to the heterogeneity of stroke survivors and their deficits. However, it was not our intention that one or two app solutions should be applicable for all stroke survivors and in all rehabilitation settings and contexts. Nevertheless, by involving six different stroke rehabilitation settings in the development of the content in the apps, some context-specific considerations have been considered.

## Conclusion

Using a structured and facilitated six-stages EBCD approach supported the development of the content in two app solutions “Mit Sygehus” and “Genoptræn.dk” to support a person-centred and empowering stroke rehabilitation. Using open-ended questions, pairing stroke survivors and significant others with a therapist they knew, and ongoing facilitation during workshops, showed to have a positive impact on participants’ sharing, engaging, learning, and collaboration in the EBCD approach. Using an EBCD approach may ultimately also support the subsequent implementation.

### Supplementary Information

**Additional file 1.** The GRIPP2 reporting checklist on patient and public involvement in research.

## Data Availability

Not applicable.

## Abbreviations

OTs: Occupational therapists
PTs: Physiotherapists
ICT: Information and communication technologies
Apps: Mobile phone and tablet applications
EBCD: Experience-based co-design
GT: Grounded theory
