# Supplementary material for: Using experience-based co-design to develop mobile/tablet applications to support a person-centred and empowering stroke rehabilitation
Source: Res Involv Engagem. 2023 Aug 24;9:69. doi: 10.1186/s40900-023-00472-z (PMC10463694; doi:10.1186/s40900-023-00472-z)
Supplement: Supplementary file 1 — Additional file 1. The GRIPP2 reporting checklist on patient and public involvement in research. [file 40900_2023_472_MOESM1_ESM.docx]

## Additional file: GRIPP2 reporting checklists: tools to improve reporting of patient and public involvement in research

| **Section and topic** | **Item** | **Reported on page NO** |
| --- | --- | --- |
| **1: Aim** | **Report the aim of PPI in the study**  To involve stroke survivors, significant others, therapist from diverse rehabilitation settings, patient organizations, app-developers and researchers as partners in co-designing two app-solutions to support person-centred and empowering stroke rehabilitation, based on the needs of involved stakeholders. | Page 5 |
| **2: Methods** | **Provide a clear description of the methods used for PPI in the study**  A structured, facilitated EBCD approach comprising six stages was used to co-produce a service that aimed to address the priorities and needs of all relevant end-users.  Stroke survivors (n=23), significant others (n=18), occupational therapists (n=12), physiotherapists (n=9), representative of a patient organization (n=1), application developers (n=3) and researchers (n=2) were recruited throughout the research stages of the development. Participant were engaged in identifying needs and unmet needs in current stroke rehabilitation, in prioritizing the content in the two app-solutions, in developing and adjusting the content in the app-solutions to match needs and to test the solutions before implementation in 10 cases throughout the stroke rehabilitation trajectory.  Consensus was not the aim in the workshops used in the development stage, rather diversity in experiences and needs.  Data were collected by interviews, participant observations, notes on “flip sheets” and written feedback on the content in the apps and on the instruction pages. Data were analyzed descriptively and with a constructivist grounded theory analysis. | Page 2, 6,7,8, 11, 17, 18 and table 1 |
| **3: Study results** | **Outcomes—Report the results of PPI in the study, including both positive and negative outcomes**  By involving stroke survivors, significant others, therapists from diverse rehabilitation settings, patient organizations, app-designers, and researchers, we co-designed two app-solutions to support a person-centred and empowering stroke rehabilitation, based on the needs and wishes of all stakeholders. Stroke survivors and significant others experienced that their voices were heard throughout the co-design process, and that their contribution was considered important and valued. | Page 16 |
| 4: Discussion and conclusions | **Outcomes—Comment on the extent to which PPI influenced the study overall. Describe positive and negative effects**  In this study end-users were involved throughout the process of developing app solutions to enable a person-centred and empowering stroke rehabilitation. To maintain momentum during the workshops’ different stages, actions and activities, we learned that facilitation was important – supplied by researchers, the therapists and app-developers.  In the development process, the participants prioritized the “knowledge” module and “training” module as of greatest importance, thus the content in the app-solutions were prioritized around these prioritized needs.  Also, easy and user-friendly solutions were developed, and tested by participants before continuing to an implementation and testphase.  Despite knowledge on unequal power dynamics in co-production designs participants’ engagement in this development process was associated with positive experiences, including learning together and a feeling of being listened to. | Page 24 |
| 5: Reflections/critical perspective | **Comment critically on the study, reflecting on the things that went well and those that did not, so others can learn from this experience.**  The first author introduced the background for conducting the workshops, so that participant had a clear understanding of the purpose of these workshops, and that their role was important.  The stroke survivors and significant others were paired with a therapist they knew, so that they would feel comfortable and motivated to contribute equally to the workshops. Being paired with therapists they knew had a positive effect on sharing their experiences.  Stroke survivors’ and significant others contribution and voiced were valued, og one PT even suggested that more stroke survivors and significant others could have been included to get more perspectives  Data analysis from participants’ inputs on artefacts such as “flip-sheets”, and their prioritization, facilitated the discussion on how to proceed to the next stage of EBCD.  Questions from the therapists and/or the two facilitators (researchers), such as: “what content would have supported you in this module?”, “would you use this module?” and “if we need to learn from this, what would you suggest be done differently” made it possible to show sincere interest in making stroke rehabilitation better for participants and supported their active engagement.  Despite different perspectives and experiences participants co-constructed their prioritization of each module through sharing their perspectives.  To maintain momentum during the workshops’ different stages, actions and activities, we learned that facilitation was important – supplied by researchers, the therapists and app-developers.  Stage 5 and 6 in the EBCD process provided smaller adjustment before implementation and test phase: this stage revealed the need for smaller adaptions; for example, the duration of the recorded videos was made visible, and all engaged therapists had access to the stroke survivors’ “training” module, to be able to access assigned exercises and adapt content according to current needs of the patient (i.e., person-centred rehabilitation). Thus, stage 5 and 6 of the EBCD showed to be valuable for bringing more useable app solutions to the next stage. Especially, the need for written instruction on how to download and use the app solutions were valuable input to make implementation more successful. | Page 11, 13, 15, 16, 24 |
